# Prophylactic antibiotics on patients with cirrhosis and upper gastrointestinal bleeding: A meta-analysis

**DOI:** 10.1371/journal.pone.0279496

**Published:** 2022-12-22

**Authors:** Yanying Gao, Baoxin Qian, Xu Zhang, Hua Liu, Tao Han

**Affiliations:** 1 Department of Gastroenterology, The Third Central Hospital of Tianjin, Tianjin Key Laboratory of Extracorporeal Life Support for Critical Diseases, Artificial Cell Engineering Technology Research Center, Tianjin Institute of Hepatobiliary Disease, Tianjin, P.R. China; 2 Department of Gastroenterology, People’s Hospital Affiliated to Nankai University of Tianjin, Tianjin, P.R. China; University of Malaya Faculty of Medicine, MALAYSIA

## Abstract

**Objective:**

To evaluate the effect of different prophylactic antibiotic treatments for cirrhosis patients with upper gastrointestinal bleeding (UGIB) and to investigate whether prophylactic antibiotics are equally beneficial to reducing the risk of adverse outcomes in A/B with low Child-Pugh scores.

**Methods:**

Relevant studies were searched via PubMed, Embase, Cochrane Library, Web of Science, China National Knowledge Internet (CNKI), Wanfang, and VIP databases up to July 16, 2021. The heterogeneity test was conducted for each outcome measuring by I^2^ statistics. Subgroup analysis was performed regarding antibiotic types. Relative risk (RR) and 95% confidence interval (CI) were used to evaluate prophylactic antibiotics on the risk of adverse outcomes in cirrhosis patients with UGIB.

**Results:**

Twenty-six studies involving 12,440 participants fulfilled our inclusion criteria. Antibiotic prophylaxis was associated with a reduced overall mortality (RR: 0.691, 95%CI: 0.518 to 0.923), mortality due to bacterial infections (RR: 0.329, 95%CI: 0.144 to 0.754), bacterial infections (RR: 0.389, 95%CI: 0.340 to 0.444), rebleeding (RR: 0.577, 95%CI: 0.433 to 0.767) and length of hospitalization [weighted mean difference (WMD): -3.854, 95%CI: -6.165 to -1.543] among patients with UGIB. Nevertheless, prophylactic antibiotics may not benefit to A/B population with low Child-Pugh scores. In our subgroup analysis, quinolone, beta-lactams alone or in combination reduced adverse outcomes in cirrhosis patients with UGIB.

**Conclusion:**

Administration of antibiotics was associated with a reduction in mortality, bacterial infections, rebleeding, and length of hospitalization. Quinolone, beta-lactams alone or in combination can be used in cirrhosis patients with UGIB. Nevertheless, targeted efforts are needed to promote the appropriate use of antibiotics among patients with cirrhosis and UGIB.

## Introduction

Cirrhosis is the 10th major cause of death worldwide, which is characterized by an increased portal inflow secondary to splanchnic arterial vasodilatation and high portal outflow resistance, both leading to portal hypertension [1, 2]. Upper gastrointestinal bleeding (UGIB) is a serious complication of cirrhosis [3], which most frequently caused by gastroesophageal varices development (65%-70%), isolated gastric varices (10%-15%), or less frequently portal hypertensive gastropathy [4]. Reports showed that about one-third of cirrhotic patients had gastroesophageal variceal bleeding (GOVB) and around 20% had peptic ulcer bleeding during follow-up [2, 5]. Bacterial infections during or immediately after bleeding episodes are associated with severe complications, which were related to the substantial medical expenses and high mortality [6, 7]. UGIB is one of the leading causes of hospital admission [3]. The survival of patients after an episode of UGIB has recently improved due to the standardization in supportive management and the advances in the treatment of portal pressure and endoscopic techniques [4, 8]. However, the management of UGIB in patients with cirrhosis remains a clinical challenge.

Current clinical guidelines recommend that antibiotic prophylaxis should be instituted for cirrhotic patients with UGIB [9, 10], with the advantages of decreasing not only bacterial infections but also mortality [6, 11, 12]. Despite the positive impact of antibiotic prophylaxis on the outcomes of cirrhotic patients with UGIB, there is scant data concerning the effect of different types of antibiotic prophylaxis on the outcomes of cirrhotic patients. To select the optimal prophylactic antibiotics in cirrhotic patients with UGIB patients, stratification of the antibiotic prophylaxis should be performed. In addition, patients with varying severity of cirrhosis may have disparate levels of risk of complications during UGIB [13, 14]. In patients with Child-Pugh class A cirrhosis and UGIB, a low risk of bacterial infection and mortality has been reported [15]. A balance of the risks and benefits of routine and indistinctive prophylactic antibiotic use in all patients with cirrhosis and UGIB is essential.

Herein, this meta-analysis was to explore the effect of different types of prophylactic antibiotics on cirrhosis patients with UGIB and determine whether antibiotics are equally beneficial in reducing the risk of adverse outcomes across different cirrhotic patients.

## Methods

### Search strategy

From inception to July 16, 2021, relevant studies were identified by literature in comprehensive databases including PubMed, Embase, Cochrane Library, Web of Science, China National Knowledge Internet (CNKI), Wanfang, VIP. Search strategies from PubMed database were as follows: (“Liver Cirrhosis” OR “Hepatic Cirrhosis” OR “Cirrhosis, Hepatic” OR “Cirrhosis, Liver” OR “Fibrosis, Liver” OR “Liver Fibrosis”) AND (“upper gastrointestinal bleeding” OR “Gastrointestinal Hemorrhage” OR “Hemorrhage, Gastrointestinal” OR “Gastrointestinal Hemorrhages” OR “Hematochezia” OR “Hematochezias”) AND (“Anti-Bacterial Agents” OR “Agents, Anti-Bacterial” OR “Anti Bacterial Agents” OR “Antibacterial Agents” OR “Agents, Antibacterial” OR “Antibacterial Agent” OR “Agent, Antibacterial” OR “Anti-Bacterial Compounds” OR “Anti Bacterial Compounds” OR “Compounds, Anti-Bacterial” OR “Anti-Bacterial Agent” OR “Agent, Anti-Bacterial” OR “Anti Bacterial Agent” OR “Anti-Bacterial Compound” OR “Anti Bacterial Compound” OR “Compound, Anti-Bacterial” OR “Bacteriocidal Agents” OR “Agents, Bacteriocidal” OR “Bacteriocidal Agent” OR “Agent, Bacteriocidal” OR “Bacteriocide” OR “Bacteriocides” OR “Anti-Mycobacterial Agents” OR “Agents, Anti-Mycobacterial” OR “Anti Mycobacterial Agents” OR “Anti-Mycobacterial Agent” OR “Agent, Anti-Mycobacterial” OR “Anti Mycobacterial Agent” OR “Antimycobacterial Agent” OR “Agent, Antimycobacterial” OR “Antimycobacterial Agents” OR “Agents, Antimycobacterial” OR “Antibiotics” OR “Antibiotic”).

### Inclusion and exclusion criteria

Inclusion criteria were performed according to the PICOS principles: (1) population (P) patients diagnosed with cirrhosis complicated with UGIB; (2) intervention and control (I) and (C): patients treated with prophylactic antibiotics were regarded as the experimental group, and patients without prophylactic antibiotics treatment were served as the control group; (3) outcomes (O): overall mortality, mortality due to bacterial infections/gastrointestinal hemorrhage/liver failure/septic shock/multiple organs failure, bacterial infections, re-bleeding, hospitalization days, length of stay in the intensive care unit (ICU); (4) study design (S): randomized controlled trials (RCTs) and cohort studies; (5) literature published in English and Chinese.

Exclusion criteria were: (1) animal experiments; (2) studies in which data are incomplete or unable to be extracted; (3) conference abstracts, academic dissertations, case reports, letters, editorial materials, meta-analyses, and reviews.

### Methodological quality assessment and data extraction

The modified Jadad scale was used to assess the quality of studies [16], using the risk assessment of bias tool from the Cochrane Collaboration’s tool, regarding the following four aspects: 1) methods for generating random series (0–2 points); 2) randomization concealment (0–2 points); 3) blind method (0–2 points); 4) assessment of withdrawal (0–1 points). A final score of 1–3 was regarded as low quality and 4–7 was regarded as high quality.

From all relevant articles, we extracted authors’ names, year of publication, country, intervention, age, gender, assessment of cirrhosis, clinical outcome. Two authors (YYG and BXQ) independently extracted the data from included studies. Any disagreements were resolved by discussion with a third author (TH).

### Statistical analysis

Relative risk (RR) and hazard ratio (HR) were used as effect indicators for categorical data. Continuous data were analyzed calculating weighted mean difference (WMD), and the effect size was expressed by 95% confidence interval (CI). The heterogeneity of effects across studies was evaluated by I^2^ tests for heterogeneity. When the heterogeneity statistic I^2^≥25%, random effects model analysis was performed, otherwise, fixed effects model analysis was applied. *P*<0.05 was considered statistically significant. When the difference was statistically significant and I^2^≥25%, subgroup analysis was performed regarding antibiotic types. Sensitivity analysis was performed for all outcomes. Begg’ test was examined to evaluate the potential for publication bias. When publication bias occurred, the “trim-and-fill method” was adopted to adjust publication bias. Software Stata 15.1 (Stata Corporation, College Station, TX, USA) was used for statistical analysis.

## Results

### Literature search and characteristics of studies

**Fig 1:**
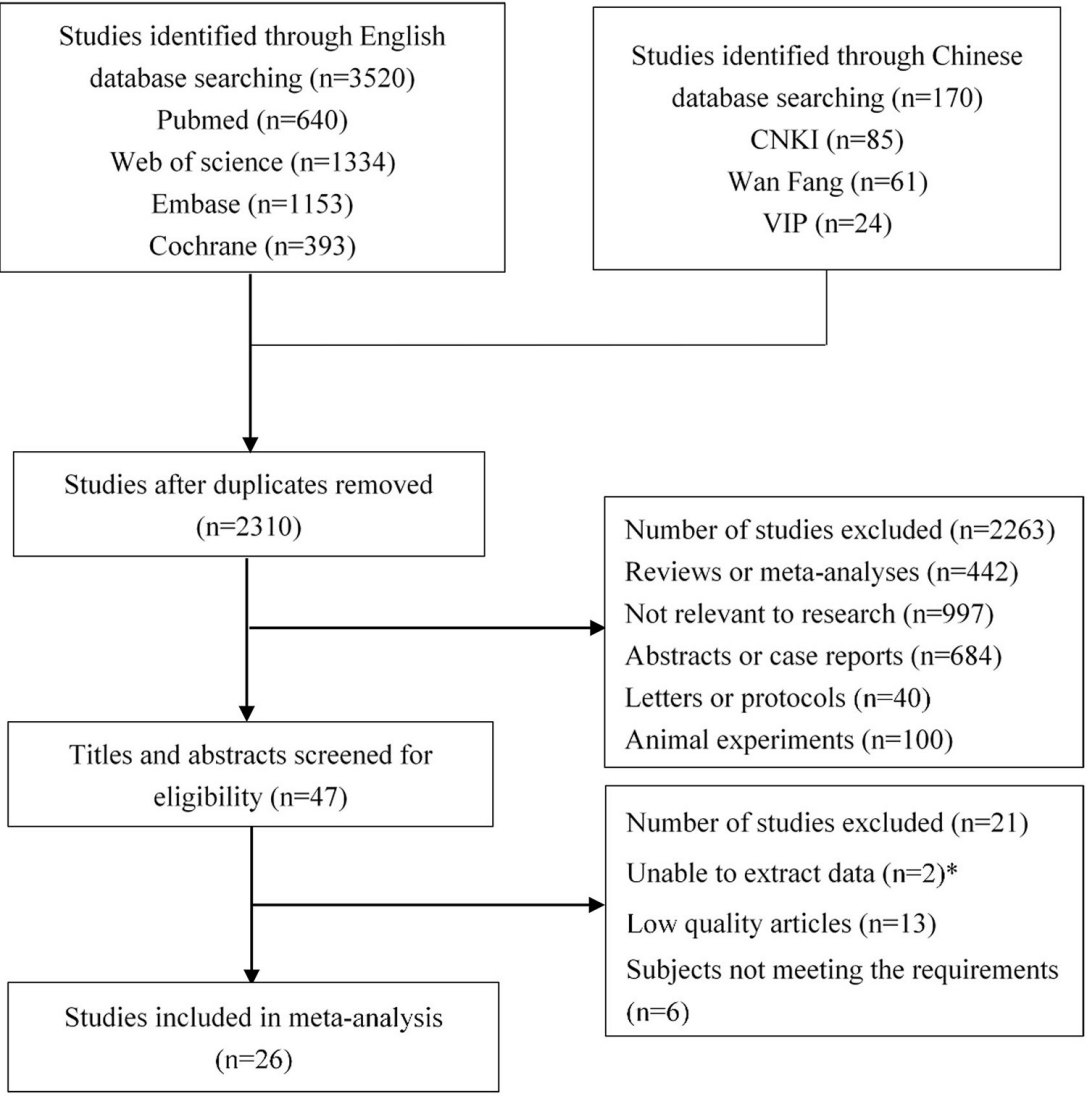
The flowchart of the literature selection.

The flowchart of the literature selection is summarized in Fig 1. Using this searching strategy, 3,690 studies were retrieved, 2,310 studies were left after duplicates removing, of which 47 articles were assessed after titles and abstracts screening. Eventually, 26 [6, 7, 14, 17–39] of these were identified, including 5,783 patients in the experimental group and 6,657 in the control group. The characteristics of each study are shown in Table 1.

**Table 1:** Baseline characteristics of included studies.

Author	Year	Country	Groups	Intervention	n	Age (years)	Male/female	Ascites	HCC	Etiology of cirrhosis	Child-Pugh A/B/C	Child-Pugh score	Clinical outcomes
Soriano	1992	Spain	T	Oral norfloxacin 800 mg ⁄ day during 7 days	60	61.0 ± 12.6	31/29	15	5	alcoholic:33, other:27	19/30/11		Overall mortality、mortality due to bacterial infections/gastrointestinal hemorrhage/liver failure、Bacterial infections、Hospitalization (days)
			C	No antibiotic prophylaxis	59	58.3 ± 13.5	30/29	14	2	alcoholic:34, other:25	21/25/13		
Rolando	1993	UK	T	Intravenous imipenem + cilastin,500 mg before and after the sclerotherapy	47	54 (20–76)	30/17	30	NA	NA	NA		Overall mortality、Bacterial infections
			C	Intravenous dextrosesaline solution	50	46 (18–84)	24/26	35	NA	NA	NA		
Selby	1994	Australia	T	Intravenous cefotaxime, 1 g immediately before sclerotherapy	19	58.9 ± 14.2	15/4	5	NA	Alcohol:11,HBV:2,HCV:3,Other:3	4/8/7		Overall mortality、Bacterial infections
			C	No antibiotic prophylaxis	20	49.5 ± 10.7	13/7	7	NA	Alcohol:12,HBV:1,HCV:4,Other:3	4/10/6		
Blaise	1994	France	T	Intravenous + oral ofloxacin,400 mg ⁄ day, 10 days;amoxicillin + clavulanic acid(bolus, 1 g) before each endoscopy procedure	46	52 ± 11	33/13	22	NA	alcoholic:41, other:5	0/11/35		Overall mortality、mortality due to gastrointestinal hemorrhage/septic shock、Bacterial infections、Recurrence of hemorrhage、Length of stay in ICU (d)
			C	No antibiotic prophylaxis	45	54 ± 9	36/9	17	NA	alcoholic:39, other:6	0/9/36		
Pauwels	1996	France	T	Intravenous + oral ciprofloxacin 400 mg ⁄ day, amoxicillin-clavulanic acid 3 g ⁄ day, until 3 days after cessation of haemorrhage	30	51 ± 2	14/16	16	NA	alcoholic:27, other:3	2/3/25		Overall mortality、mortality due to septic shock/gastrointestinal hemorrhage/liver failure、Bacterial infections、Recurrence of hemorrhage、Length of stay in ICU (d)
			C	No antibiotic prophylaxis	34	53 ± 3	24/10	13	NA	alcoholic:21, other:13	0/10/24		
Zacharof	1997	Greece	T	Oral ciprofloxacin 500 mg ⁄ day during 7 days	35	NA	NA	NA	NA	NA	NA		Overall mortality、Bacterial infections
			C	No antibiotic prophylaxis	30	NA	NA	NA	NA	NA	NA		
Hsieh	1998	Taiwan	T	Oral ciprofloxacin, 1 g ⁄ day, 7 days	60	58 ± 14	47/13	28	29	HBV/HCV:53,alcoholic:6,other:1	5/33/22		Overall mortality、Bacterial infections、Recurrence of hemorrhage、Hospitalization (days)
			C	Placebo	60	62 ± 13	42/18	30	19	HBV/HCV:48,coholic:9,other:3	6/31/23		
Hou	2004	Taiwan	T	Intravenous ofloxacin 200 mg b.d. for 2 days followed by oral ofloxacin 200 mg b.d. for 5 days	59	60.02 ± 13.92	43/16	33	16	Viral/alcohol/mixed/others: 29/6/10/14	10/35/14	8.54 ± 1.90	Overall mortality、mortality due to bacterial infections/gastrointestinal hemorrhage/liver failure/multiple organs failure、Bacterial infections、Recurrence of hemorrhage
			C	No antibiotic prophylaxis	61	59.39 ± 14.85	48/13	29	14	Viral/alcohol/mixed/others:34/10/10/7	19/29/13	7.90 ± 2.04	
Jun	2006	Korea	T	Intravenous cefotaxime 2 g t.d.s. for 7 days	62	54.7 ± 10.1	54/8	34	16	Esophageal/gastric varices:52/11		8.7 ± 1.9	Overall mortality、mortality due to bacterial infections/gastrointestinal hemorrhage/liver failure/multiple organs failure、Bacterial infections、Recurrence of hemorrhage、Hospitalization (days)
			C	No antibiotic prophylaxis	58	54.2 ± 11.9	56/2	33	10	Esophageal/gastric varices:50/8		8.3 ± 2.1	
Tandon	2015	Canada	T	Antibiotic prophylaxis	206	55.0 ± 11.7	154/52	NA	14	Alcohol or HCV:121	16/97/93	9.4 ± 2.1	Overall mortality、Bacterial infections、Recurrence of hemorrhage
			C	No antibiotic prophylaxis	175	56.2 ± 13.4	114/61	NA	11	Alcohol or HCV:92	30/94/51	8.5 ± 2.0	
Moon	2016	USA	T	3rd generation cephalosporins 54.5%, Fluoroquinolones 31.9%, Penicillins 2.9%, Aminoglycosides 0.8%, other 9.9%	4210	60.0 ± 8.5	4100/110	1003	405	HCV:2103,Alcohol:1597,HBV:71,Non-alcoholic fatty liver disease:354,Other:85	NA		Overall mortality
			C	No antibiotic prophylaxis	4445	61.2 ± 9.6	4333/112	459	290	HCV:1895,Alcohol:1790,HBV:72,Non-alcoholic fatty liver disease:545,Other:143	NA		
Chang	2020	Taiwan	T	19 received cefazolin, 4 received cefazolin plus gentamicin, 6 received cefuroxime, and 44 received ceftriaxone	73	55.22 ± 12.46	57/16	33	19	HBV:22.HCV:28,BC:8,NBNC:15	18/55/0	7.34 ± 1.20	Overall mortality、Bacterial infections、Recurrence of hemorrhage
			C	No antibiotic prophylaxis	840	59.86 ± 13.15	592/248	344	287	HBV:173.HCV:404,BC:77,NBNC:186	239/601/0	7.32 ± 1.21	
Qin	2002	China	T	Intravenous infusion of ofloxacin (200mg, twice a day) for 7 consecutive days	50	51 ± 12	42/8	40	NA	hepatitis:38, alcoholic:8, other:4	1/9/40		Overall mortality、Bacterial infections、Recurrence of hemorrhage、Hospitalization (days)
			C	No use of antibiotic prophylaxis	50	53 ± 10	43/7	42	NA	hepatitis:40, alcoholic:7, other:3	2/10/38		
Wu	2002	China	T	On the day of admission, ciprofloxacin or piperacillin was given intravenously, 0.4 g of ciprofloxacin or piperacillin 6–8 g was instilled twice a day, until 3 days after the bleeding stopped	54	47.6 ± 21.9	44/10	47	NA	HBV	NA	9.3 ± 2.7	Overall mortality、mortality due to bacterial infections、Bacterial infections
			C	No antibiotics were given after admission	48	45.1 ± 26.3	38/10	41	NA	HBV	NA	9.1 ± 2.2	
Ma	2007	China	T	Ceftriaxone antibiotics were given immediately after admission, 2g once / 12h, until 3 days after the bleeding stopped	21	40.4 ± 17.4	33/9	21	NA	NA	NA		Bacterial infections
			C	Conventional treatment	21			21	NA	NA	NA		
Luo	2008	China	T	Quinolones or cephalosporin third-generation antibiotics were given for 1 week after admission	60	40.3 (21–73)	68/52	NA	NA	Alcohol:4,HBV:82,HCV:34	B/C		Overall mortality、Bacterial infections
			C	No antibiotics	60			NA	NA				
Wang	2015	China	T1	Anti-infection treatment with ceftriaxone sodium after admission	60	42–69	42/18	NA	NA	hepatitis:48, alcoholic:11, other:1			Overall mortality、Bacterial infections、Recurrence of hemorrhage
			T2	Levofloxacin was used for anti-infection treatment for 7 days	60		40/20	NA	NA	hepatitis:45, alcoholic:14, other:1			
			C	Antibiotics were not given on admission	60		38/22	NA	NA	hepatitis:47, alcoholic:13, other:0			
Liu	2015	China	T	After admission, antibiotics were used on the basis of conventional treatment to prevent infection, ceftriaxone sodium was injected intravenously 1g, 1 time/d; for those who are allergic to it (2 cases in total), levofloxacin intravenously injected 0.4g, 1 time/d; prevention Infection treatment 7days	210	NA	160/50	NA	NA	HBV:147,HCV:25,Alcohol:14,autoimmune hepatitis:11,other:3	123/66/21		Bacterial infections、Recurrence of hemorrhage
			C	No antibiotics	173	NA	134/39	NA	NA	HBV:138,HCV:12,Alcohol:5,autoimmune hepatitis:2,other:16	115/38/20		
Zheng	2017	China	T	After admission, cefotaxime sodium 2.0g was given intravenously, twice a day, for 7 days	60	56.5±12.4	51/9	NA	NA	hepatitis:45, alcoholic:14, other:1	44/13/3		Overall mortality、Bacterial infections、Recurrence of hemorrhage
			C	No antibiotics were administered on admission	60		52/8	NA	NA	hepatitis:47, alcoholic:13, other:0	45/13/2		
Na	2017	China	T	Add human cefotaxime sodium 2.0g intravenously to the treatment plan of the control group, bid, 3 days after the bleeding stops	22	45.5 ±2.2	10/12	NA	NA	hepatitis:12, alcoholic:8, other:2			Overall mortality、Bacterial infections、Recurrence of hemorrhage、Hospitalization (days)
			C	Routine treatment was given first, pituitrin or octreotide were given, respectively, and intravenous drops were dropped until the bleeding stopped for 4 days. At the same time, auxiliary therapy of blood transfusion, electrocardiogram, hemostasis and oxygen inhalation was performed	28	47.5 ± 2.3	12/16	NA	NA	hepatitis:15, alcoholic:10, other:3			
Zhang	2018	China	T	After admission, on the basis of the control group, intravenous infusion of ceftriaxone disodium, 1g/ time, once a day, for cephalosporin allergy, intravenous infusion of levofloxacin, 0.4g/ time, once a day, continuous application of antibiotics for 1 week	52	45. 19 ± 5. 34	33/19	NA	NA	hepatitis:50, alcoholic:2			Overall mortality、Bacterial infections、Recurrence of hemorrhage、Hospitalization (days)
			C	Conventional treatment	52		35/17	NA	NA	hepatitis:49, alcoholic:3			
Wang	2014	China	T	Prophylactic use of antibiotics (piperacillin or ciprofloxacin) in addition to conventional treatment, piperacillin 5.0–9.0g/d, twice daily iv or ciprofloxacin 0.5g/d, twice daily IV, 3 days as a course of treatment	60	49±3.6	36/24	NA	NA	hepatitis:38, alcoholic:19, schistosomal cirrhosis:2,other:1			Overall mortality、Bacterial infections、Recurrence of hemorrhage、Hospitalization (days)
			C	Conventional treatment	60		31/29	NA	NA	hepatitis:38, alcoholic:20, schistosomal cirrhosis:1,other:1			
Dong	2015	China	T	On the basis of conventional treatment, antibiotics were given to patients after blood, urine and ascites culture specimens were collected (levofloxacin 0.4g intravenously, once per day, or cefotaxime sodium 4-6g intravenously, once per 12h, 5 days as a course	49	NA	NA	NA	NA	NA	C		Overall mortality、Bacterial infections、Hospitalization (days)
			C	Conventional treatment	50	NA	NA	NA	NA	NA			
Peng	2017	China	T	On the basis of conventional treatment, intravenous infusion of cephalosporin iii antibiotics or quinolones antibiotics was performed according to patients’ allergic constitution	30	40.49 ± 6.32	18/12	NA	NA	HBV:19,HCV:8,Alcohol:3			Overall mortality、Bacterial infections
			C	Routine treatments such as blood transfusion, hemostasis and liver protection were given	30	41.25 ± 7.15	17/13	NA	NA	HBV:20,HCV:8,Alcohol:2			
Qin	2020	China	T	Prophylactic antibiotic therapy was given immediately after admission: norfloxacin 400mg orally, once a day; Ceftriaxone sodium for injection 1g was given intravenously, once a day; The third generation cephalosporin was selected for treatment for 5–7 days	45	54.76±8.69	26/19	NA	NA	NA	19/17/9		Overall mortality、Bacterial infections、Recurrence of hemorrhage
			C	Conventional treatment	45	56.31±8.83	29/16	NA	NA	NA	16/14/15		
Xu	2021	China	T	On the basis of conventional treatment, patients were given cefoperazone sulbactam 3.0g intravenous infusion, twice a day; The allergic patients were treated with 0.4g levofloxacin hydrochloride injection 3.0g intravenously, twice a day	43	42.52±9.83	23/20	NA	NA	NA			Overall mortality、Bacterial infections、Recurrence of hemorrhage
			C	Conventional treatment	43	42.55±10.15	22/21	NA	NA	NA			

**Notes:** T: patients treated with prophylactic antibiotics; C: patients not treated with prophylactic antibiotics; NA represents that data is missing; MELD: model for end-stage liver disease; HCC: hepatocellular carcinoma; HBV: hepatitis B virus; HCV: hepatitis C virus; BC: presence of both HBV and HCV; NBNC: negative for both HBV and HCV.

### Overall mortality

**Fig 2:**
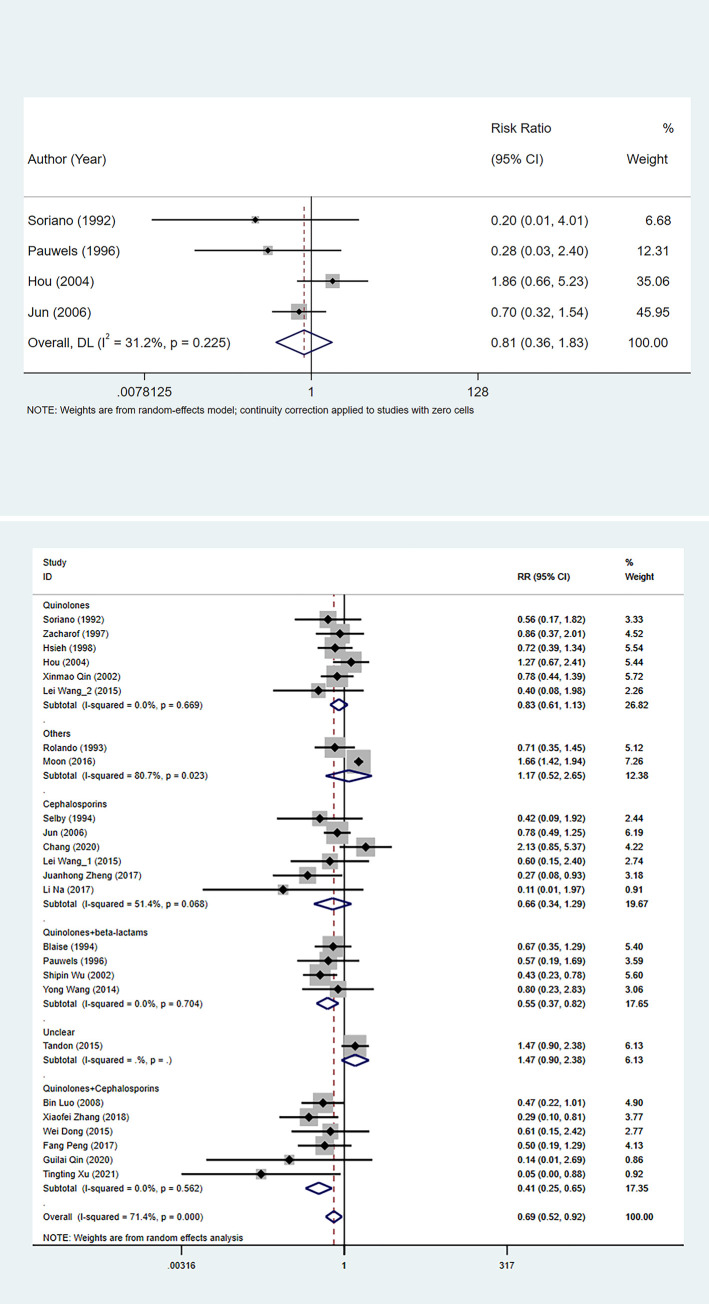
The forest plot of comparison on the overall mortality in experimental group and control group. (a) overall; (b) subgroup.

A total of 24 studies were assessed to investigate overall mortality between the experimental and control group. The heterogeneity test showed that the difference was statistically significant (I^2^ = 71.4%), so the random effects model was used. The result showed that antibiotic prophylaxis was associated with a significant reduction in overall mortality (RR: 0.691, 95%CI: 0.518 to 0.923, *P* = 0.012) (Table 2, Fig 2A). Subgroup analysis was applied based on antibiotic types. Combined quinolone and cephalosporins reduced the overall mortality of in cirrhosis patients with UGIB. Decreased overall mortality was also observed in cirrhosis patients with UGIB when quinolones were combined with beta-lactams (Fig 2B).

**Table 2:** Overall results and sensitivity analysis.

Indicators	RR/WMD (95%CI)	*P*	I^2^
Overall mortality			
**Overall**	0.691 (0.518, 0.923)	0.012	71.4
Sensitivity analysis	0.691 (0.518, 0.923)		
**Antibiotics**			
Quinolones	0.827 (0.608, 1.125)	0.227	0.0
Cephalosporins	0.663 (0.341, 1.289)	0.226	51.4
Quinolones+cephalosporins	0.406 (0.254, 0.650)	<0.001	0.0
Quinolones+beta-lactams	0.553 (0.374, 0.816)	0.003	0.0
Others	1.173 (0.519, 2.650)	0.701	80.7
Unclear	1.467 (0.904, 2.383)	0.121	NA
Mortality due to bacterial infections			
**Overall**	0.329 (0.144, 0.754)	0.009	12.7
Sensitivity analysis	0.329 (0.144, 0.754)		
Mortality due to gastrointestinal hemorrhage			
**Overall**	0.942 (0.524, 1.694)	0.842	0.0
Sensitivity analysis	0.942 (0.524, 1.694)		
Mortality due to liver failure			
**Overall**	0.811 (0.359, 1.832)	0.615	31.2
Sensitivity analysis	0.811 (0.359, 1.832)		
Mortality due to septic shock			
**Overall**	0.292 (0.050, 1.704)	0.171	0.0
Sensitivity analysis	0.292 (0.050, 1.704)		
Mortality due to multiple organs failure			
**Overall**	1.084 (0.436, 2.693)	0.863	0.0
Sensitivity analysis	1.084 (0.436, 2.693)		
Bacterial infections			
**Overall**	0.389 (0.340, 0.444)	<0.001	16.4
Sensitivity analysis	0.389 (0.340, 0.444)		
Publication bias	Z = 1.98	0.047	
Rebleeding			
**Overall**	0.577 (0.433, 0.767)	<0.001	64.5
Sensitivity analysis	0.577 (0.433, 0.767)		
Publication bias	Z = 1.07	0.284	
**Antibiotics**			
Quinolones	0.521 (0.353, 0.758)	0.001	10.8
Cephalosporins	0.517 (0.380, 0.703)	<0.001	0.0
Quinolones+cephalosporins	0.480 (0.343, 0.671)	<0.001	0.0
Quinolones+ beta-lactams	0.764 (0.264, 2.210)	0.619	82.2
Unclear	1.732 (1.133, 2.646)	0.011	NA
Hospitalization (days)			
**Overall**	-3.854 (-6.165, -1.543)	0.001	82.8
Sensitivity analysis	-3.854 (-6.165, -1.543)		
**Antibiotics**			
Quinolones	-4.115 (-8.202, -0.027)	0.049	52.5
Cephalosporins	-1.083 (-3.354, 1.188)	0.350	0.0
Quinolones+cephalosporins	-4.999 (-11.849, 1.851)	0.153	95.9
Quinolones+beta-lactams	-5.500 (-6.881, -4.119)	<0.001	NA
Length of stay in ICU (days)			
**Overall**	-0.272 (-1.545, 1.002)	0.676	89.3
Sensitivity analysis	-0.272 (-1.545, 1.002)		

**Notes:** RR: relative risk; WMD: weighted mean difference.

#### Mortality due to bacterial infections

**Fig 3:**
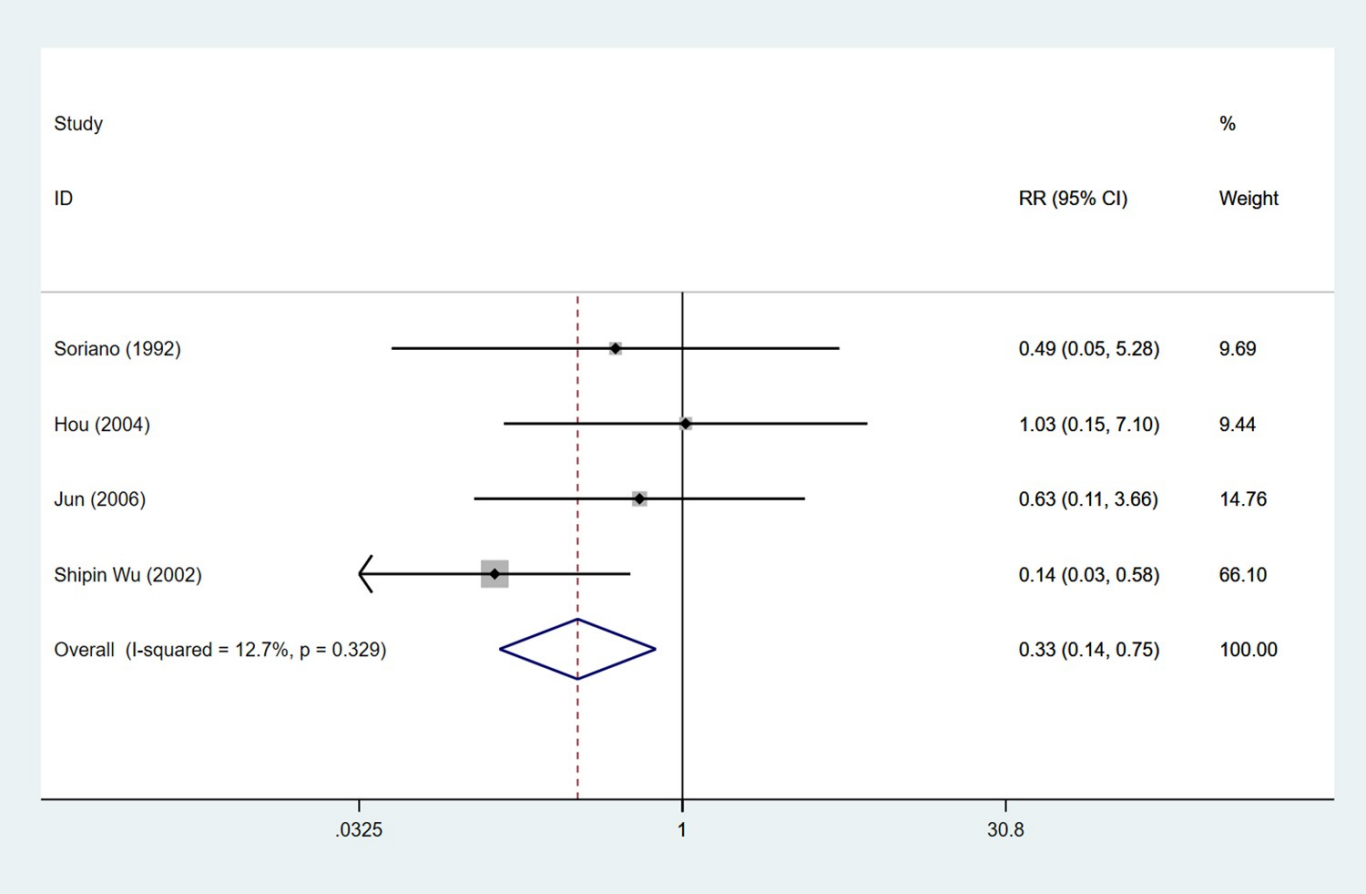
The forest plot of comparison on the mortality due to bacterial infections in experimental group and control group.

Four articles were used to evaluate mortality due to bacterial infections. The result showed a benefit of antibiotic prophylaxis over no antibiotic prophylaxis intervention regarding mortality due to bacterial infections (RR: 0.329, 95%CI: 0.144 to 0.754, *P* = 0.009) (Table 2, Fig 3).

#### Mortality due to gastrointestinal hemorrhage

Mortality due to gastrointestinal hemorrhage was assessed in 5 studies. The result could not prove a beneficial effect of antibiotic prophylaxis over mortality from bacterial infections (RR: 0.942, 95%CI: 0.524 to 1.694, *P* = 0.842) (Table 2).

### Mortality due to liver failure

Four studies investigated mortality due to liver failure. The heterogeneity test showed I^2^ = 31.2%, so the random effects model was applied. There was no significant difference in mortality due to liver failure between the experimental and control group (RR: 0.811, 95%CI: 0.359 to 1.832, *P* = 0.615) (Table 2).

#### Mortality due to septic shock

Two studies were included to assess mortality due to septic shock between experimental and control group. No significant difference between experimental and control group was observed concerning mortality due to septic shock (RR: 0.292, 95%CI: 0.050 to 1.704, *P* = 0.171) (Table 2).

#### Mortality due to multiple organs failure

Mortality due to multiple organs failure was evaluated in 2 studies. No difference in mortality from multiple organ failure was observed between the antibiotic group and the control group (RR: 1.084, 95%CI: 0.436 to 2.693, *P* = 0.863) (Table 2).

#### Bacterial infections

**Fig 4:**
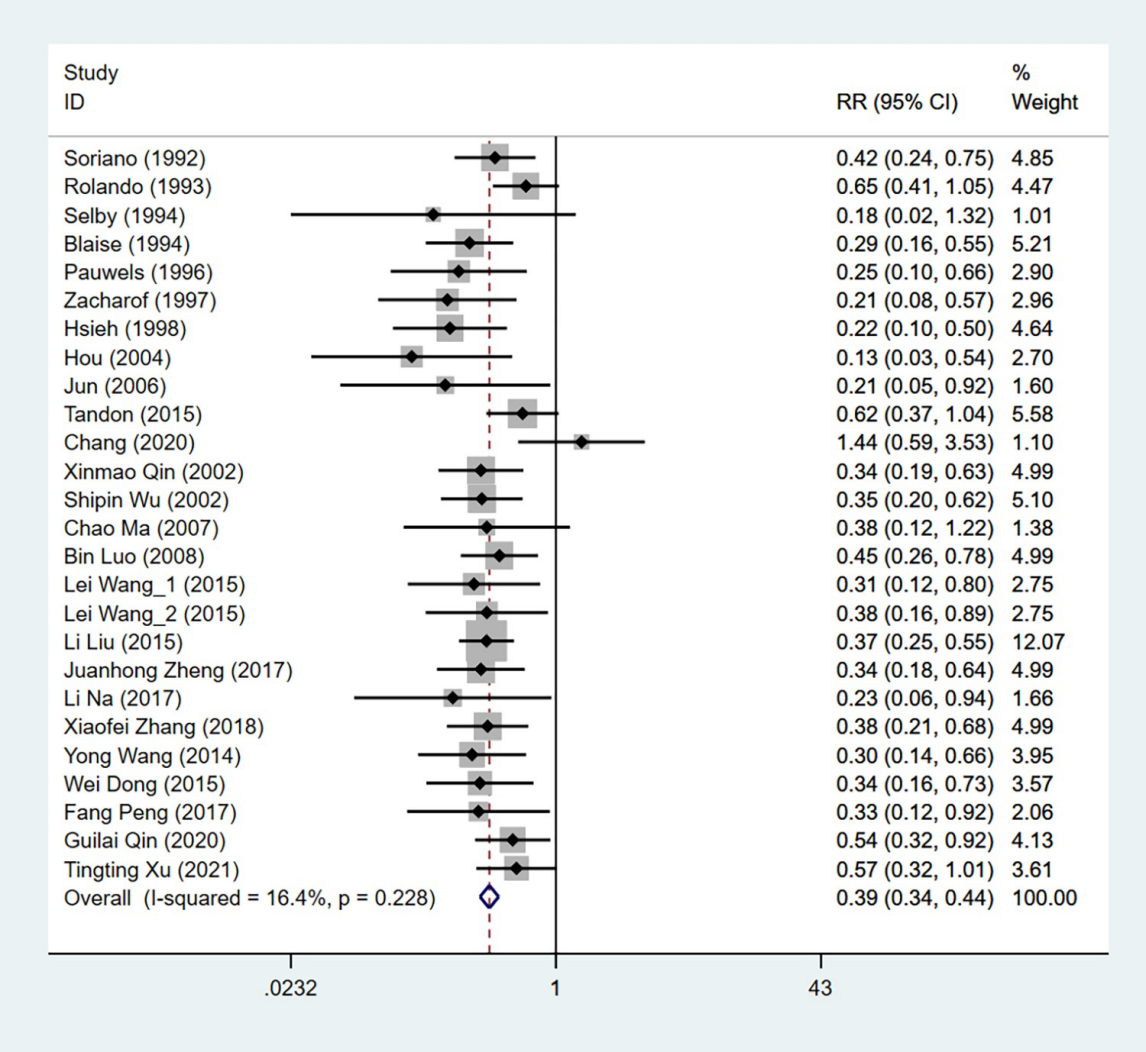
The forest plot of comparison on the bacterial infections in experimental group and control group.

Totally, 25 studies were included to evaluate the bacterial infections between 2 groups. Fixed effect model results showed that the bacterial infection rate was lower in the antibiotic prophylaxis group than in the control group (RR: 0.389, 95%CI: 0.340 to 0.444, *P*<0.001) (Table 2, Fig 4).

#### Rebleeding

**Fig 5:**
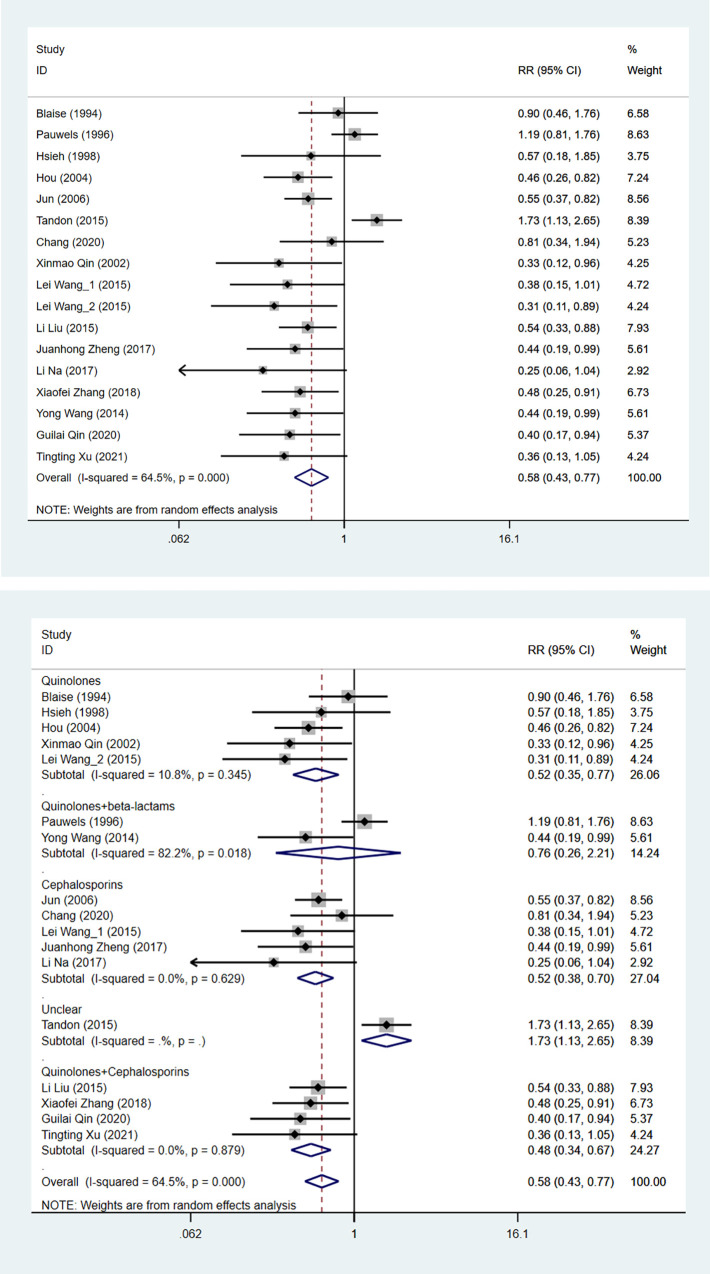
The forest plot of comparison on the rebleeding in experimental group and control group. (a) overall; (b) subgroup.

Rebleeding was investigated in 16 studies. The result reported that a decreased rate of rebleeding in the antibiotic prophylaxis group (RR: 0.577, 95%CI: 0.433 to 0.767, *P*<0.001) (Table 2, Fig 5A). Subgroup analysis was applied concerning antibiotic types. Subgroup analysis result showed that quinolone (RR: 0.521, 95%CI: 0.353 to 0.758, *P* = 0.001), cephalosporins (RR: 0.517, 95%CI: 0.380 to 0.703, *P*<0.001), quinolone in combination with cephalosporins (RR: 0.480, 95%CI: 0.343 to 0.671, *P*<0.001) reduced the rate of rebleeding (Fig 5B).

### Duration of treatment

#### Hospitalization

**Fig 6:**
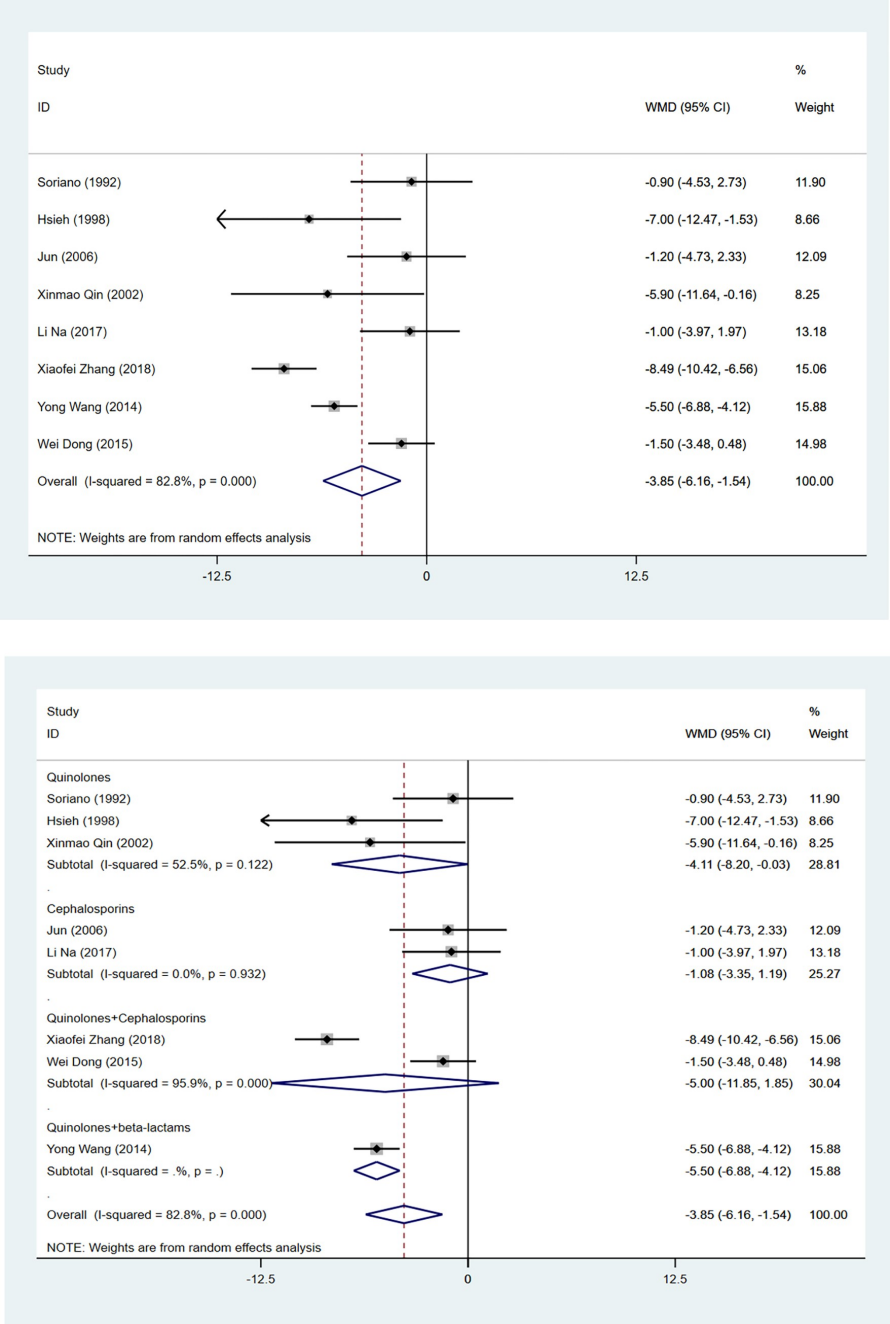
The forest plot of comparison on the hospitalization in experimental group and control group. (a) overall; (b) subgroup.

Eight studies compared hospitalization between the antibiotic group and the control group. The use of antibiotic prophylaxis reduced hospitalization in cirrhosis patients with UGIB (WMD: -3.854, 95%CI: -6.165 to -1.543, *P* = 0.001) (Table 2, Fig 6A). Subgroup analysis result demonstrated that the use of quinolones (WMD: -4.115, 95%CI: -8.202 to -0.027, *P* = 0.049), quinolone combined with beta-lactams were associated with shorter hospital stays (Fig 6B).

#### Length of stay in ICU

Two articles were used to compare the length of stay in ICU between 2 groups. The random effect model result showed that there was no difference in length of stay in ICU between the antibiotic group and the control group (WMD: -0.272, 95%CI: -1.545 to 1.002, *P* = 0.676) (Table 2).

### Child-Pugh’s score

#### Child-Pugh A/B

The effect of Child-Pugh A/B on antibiotic use was assessed in 2 studies. The result showed that antibiotic use in the Child-Pugh A/B patients had no significant effect on rates of mortality (RR: 1.819, 95%CI: 0.886 to 3.733, *P* = 0.103) and bacterial infection (RR: 0.826, 95%CI: 0.274 to 2.489, *P* = 0.735). The effect of prophylactic antibiotics for Child-Pugh A/B population is shown in Table 3.

**Table 3:** Prophylactic antibiotics for Child-Pugh A/B population.

Indicators	RR (95%CI)	*P*	I^2^
Overall mortality			
**Overall**	1.819 (0.886–3.733)	0.103	0.0
Sensitivity analysis	1.819(0.886–3.733)		
Bacterial infections			
**Overall**	0.826 (0.274–2.489)	0.735	65.9
Sensitivity analysis	0.826 (0.274–2.489)		

### Sensitivity analysis and publication bias

**Fig 7:**
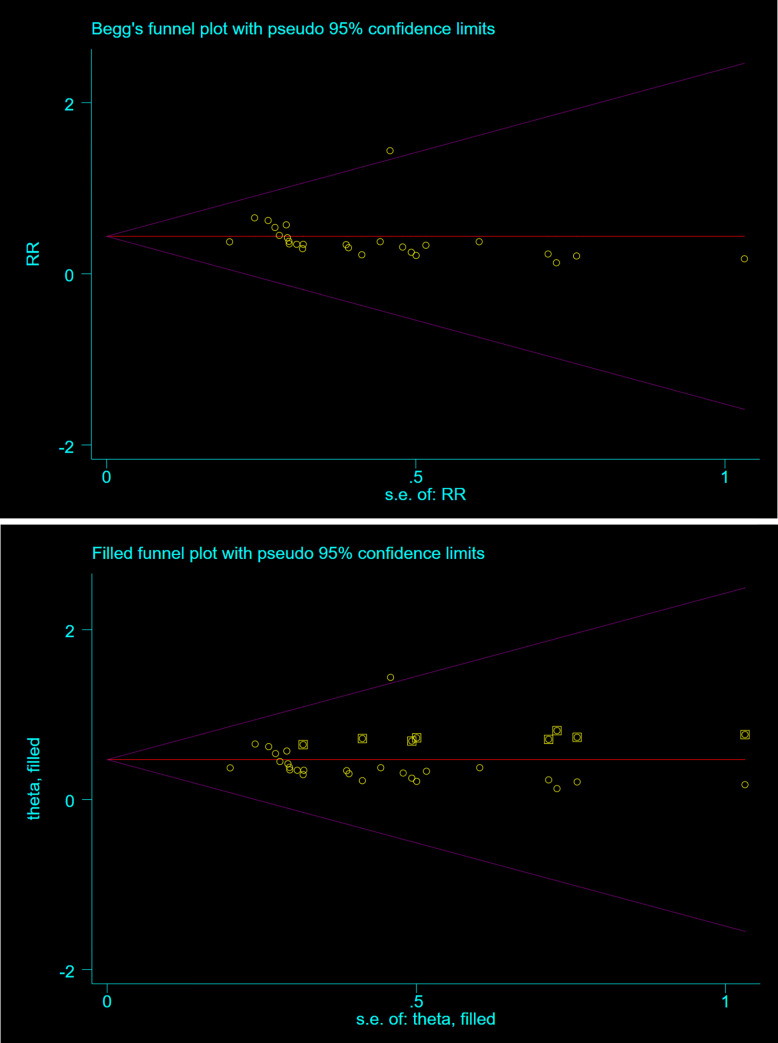
Publication bias. (a); unadjusted (b) adjusted.

The sensitivity analysis for ensemble RR of each model was carried out. Sensitivity analysis result proofs that the findings are trustworthy (Table 2). We examined the publication bias of the included studies. The result showed that publication bias existed in bacterial infections. We used the “cut-and-fill method” to correct publication bias and adjust effect size. The combined RR of the fixed effects model prevalence merger was 0.389 (95%CI: 0.340 to 0.444) before the “trim-and-fill method”. It then showed the estimated number of missing studies was 8. Then, the missing studies were included in the model, and all the studies were re-meta-analyzed. The combined prevalence in the fixed effect model was 0.470 (95%CI: 0.345 to 0.595) (Fig 7).

## Discussion

The bleeding events in cirrhosis patients were related to substantial medical expenses and high mortality [2]. The prophylactic use of oral or intravenous antibiotics has been recommended in several consensus guidelines [2, 40]. Nevertheless, studies to clarify the role of different types of antibiotic prophylaxis in cirrhosis patients with UGIB are scarce. And whether antibiotic prophylaxis is beneficial for all cirrhotic patients with UGIB is also unclear. The result of the current study proved the benefit of antibiotic prophylaxis for reductions in overall mortality and mortality due to bacterial infections among patients with UGIB. Besides, antibiotic prophylaxis was associated with reduced bacterial infections, rebleeding, and length of hospitalization. Nevertheless, prophylactic antibiotics may not benefit to A/B population with a low Child-Pugh score. In our subgroup analysis, quinolone, beta-lactams alone or in combination quinolone reduced the rate of rebleeding and shortened the length of hospital stay in cirrhosis patients with UGIB.

The benefits of antibiotic prophylaxis in cirrhosis patients with UGIB were observed in this study. Chavez-Tapia et al. [40] demonstrated that antibiotics were associated with a substantially lower 30-day all-cause mortality after adjusting for relevant confounders. Wu et al. [2] suggested that cirrhotic patients without major complications who suffered from UGIB benefited from the use of antibiotics to prevent rebleeding. A study showed that antibiotic prophylaxis reduced rates of bacterial infection, rebleeding, and mortality [41]. In addition, we found that quinolones could reduce the rate of rebleeding and shorten the length of hospital stay in cirrhosis patients with UGIB. However, some studies have presented evidence suggesting that oral quinolone administration may not be the best regime for the prevention of bacterial infections in cirrhotic patients with gastrointestinal hemorrhage. The prevalence of quinolone-resistant bacteria in the fecal flora [42, 43] and the incidence of spontaneous bacterial peritonitis (SBP) [44], and other infections [45] caused by these organisms have increased substantially. In this case, other classes of antibiotics are needed. Since the discovery of benzylpenicillin in the 1920s, thousands of new penicillin derivatives and related beta-lactam classes of cephalosporins, cephamycins, monobactams, and carbapenems have been discovered. The bactericidal mechanism of killing by beta-lactams is perceived to be a major advantage in the treatment of serious infections. When these agents were threatened by the rapid emergence of beta-lactamases, beta-lactamase-stable agents were developed, as well as potent beta-lactamase inhibitors (BLIs) [46]. Beta-lactams are vital antibiotics used to treat infection in patients with cirrhosis due to their spectrum of antibacterial activity and overall safety [47]. In those with cirrhosis, 3rd generation cephalosporins (TGC) has remained for decades the standard treatment of SBP [48]. Nevertheless, the spread of multiple drug resistance (MDR) bacterial infections reduces the effectiveness of commonly used antibiotics such as third-generation cephalosporins [49]. In addition, there is currently limited therapeutic use of penicillin as monotherapy. Ampicillin, amoxicillin, piperacillin, and ticarcillin have continued to be useful, primarily as a result of their combination with an appropriate beta-lactamase inhibitor [46]. This may indicate the necessity of combined antibiotics. We found quinolone in combination with beta-lactams could be associated with reduced mortality, rebleeding rate, and hospitalization.

It is well established that the increasing Child-Pugh class is a strong predictor of bacterial infection in patients with cirrhosis [14, 50]. Excessive prescription of antibiotics has several demerits, such as multidrug resistance of bacteria, *Clostridioides difficile* infection, and increased cost [51, 52]. To avoid antibiotic overuse, some authorities have proposed a risk-stratified approach. Tandon et al. [14] suggested antibiotic prophylaxis seems unnecessary in patients with good liver function (Child-Pugh A) because they have low rates of bacterial infection and mortality even without antibiotic prophylaxis. A study [18] by Hou et al. showed that antibiotic prophylaxis effectively prevented infection and rebleeding among the most advanced cirrhosis (Child-Pugh B or C). Given this fact, we believe that the patients who will benefit from antibiotic prophylaxis should be identified, and antibiotics need not be prescribed for all bleeding cirrhotic patients. The recommendation for routine antibiotic prophylaxis in this subgroup merits expert re-evaluation and ideally confirmation in large prospective multicenter RCTs.

Potential confounding factors may affect the result of this meta-analysis. Endoscopy is the primary diagnostic and therapeutic tool for UGIB [53]. Cirrhotic patients undergoing an endoscopic procedure to control bleeding are particularly at risk for infection [6, 54]. However, a study demonstrated that the use of therapeutic endoscopy has been shown to improve better prognosis in patients who present with severe acute UGIB [55]. As there have been several exciting recent advances in the endoscopic management of UGIB [56], future studies exploring prophylactic antibiotics in cirrhosis with UGIB should consider the role of therapeutic endoscopy.

To the best of our knowledge, this study is the first meta-analysis focusing on different prophylactic antibiotics on cirrhosis patients complicated with UGIB. A comprehensive analysis of different types of mortality was performed in this study. We attempted to investigate the application of prophylactic antibiotics to low Child-Pugh scores. However, some limitations of our study must be acknowledged. Firstly, even though we adjusted for publication bias using the “trim-and-fill method, certain biases could exist, and must be cautious in extrapolating the results. Secondly, at present, there are few studies on the dose-response relationship of prophylactic antibiotics, so we did not conduct further analysis on the dose of prophylactic antibiotics. Thirdly, due to the limitations of the included study, we were unable to further classify patients with different grades of cirrhosis or assess the effect of prophylactic antibiotic use on patients with Child-Pugh’s C cirrhosis. Finally, there were too small included studies in each part. Further RCTs investigating the effect of prophylactic antibiotics on the risk of adverse outcomes in cirrhosis patients complicated with UGIB are needed.

## Conclusions

This study demonstrated that prophylactic antibiotics, especially quinolones and β -lactam alone or in combination were beneficial to cirrhosis patients with UGIB. Nevertheless, the use of antibiotic prophylaxis in Child-Pugh A/B patients with cirrhosis merits re-evaluation.

## Supporting information

S1 Checklist PRISMA 2020 checklist. (DOCX)
